# Supplementary material for: AI-Based screening for thoracic aortic aneurysms in routine breast MRI
Source: Nat Commun. 2025 Jun 12;16:5299. doi: 10.1038/s41467-025-59694-2 (PMC12162828; doi:10.1038/s41467-025-59694-2)
Supplement: Supplementary file 1 — Supplementary Information [file 41467_2025_59694_MOESM1_ESM.pdf]

AI-Based Screening for Thoracic Aortic Aneurysms in Routine Breast MRI

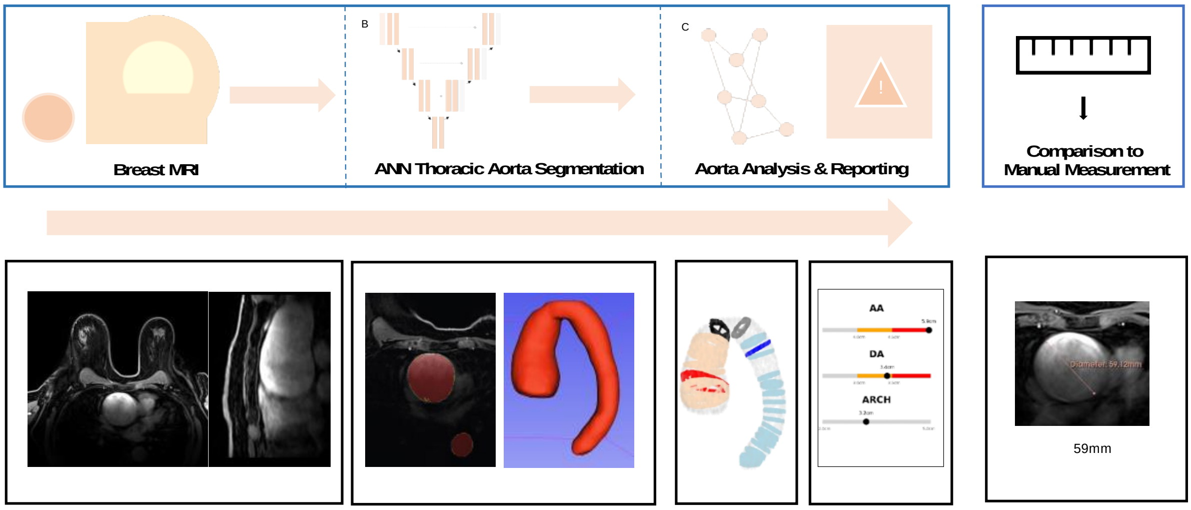

Supplementary Figure 1: Pathway of analyzing the breast MRI data.

Thoracic aortic disease screening demonstrated using routine breast MRI data (T1-weigthed contrast enhanced axial acquisition; depicted with a sagittal reconstruction of the data for illustration purposes). The ANN analyzes the data to provide a full 3D segmentation of the thoracic aorta which is then subsequently analyzed by the algorithm for the separate sections (ascending thoracic aorta, aortic arch, descending thoracic aorta). The maximum diameter of each respective section is indicated for the ascending thoracic aorta in red and the descending thoracic aorta in blue. Afterwards, the algorithm provides a report with the measurements and reference values derived from the AHA guidelines. All detected aneurysm cases in the n=5057 breast MRI examinations were manually crosschecked by a radiologists panel (D) using the inner-to-inner (I-I) approach of determining the maximum aortic diameters.

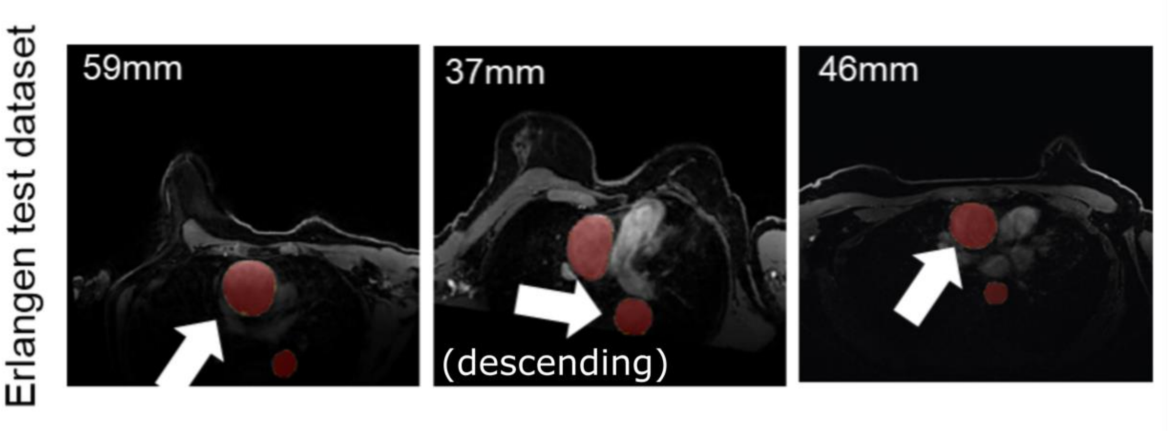

Supplementary Figure 2: Examples of thoracic aortic aneurysms detected by the ANN in the independent Erlangen test dataset on contrast enhanced (CE) T1-weighted breast MRI acquisitions.

The visualization contains axial planes adjusted to the perpendicularity of the thoracic aorta. The red areas demonstrate the aortic segmentations provided by the ANN with the presented slice indicating the location of the detected maximum thoracic aortic diameter. Case labeled with "descending" indicates an aneurysm of the descending thoracic aorta, with the rest of the cases representing detections in the ascending thoracic aorta. Diameters are given in mm. (MR images of the public datasets are not displayed in the final figure due to copyright but were available during the review process.)

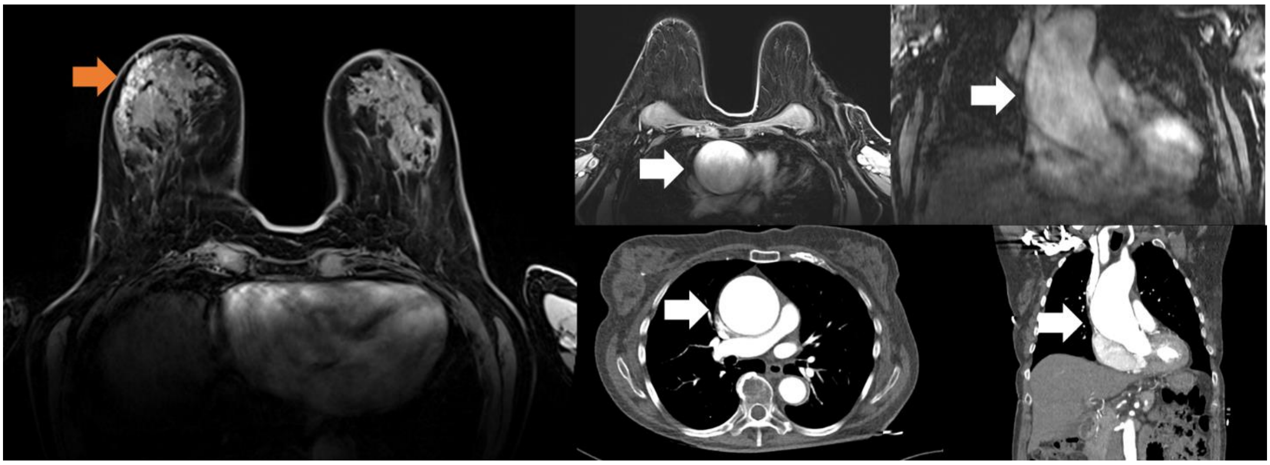

Supplementary Figure 3: Example case of a thoracic aortic aneurysm detected by the ANN pipeline and verified by computed tomography (CT).

84-year-old female patient undergoing breast MRI due to mamillary secretion in the right breast. Breast MRI indicated a BI-RADS 4 finding (left image - T1-weighted DIXON acquisition after GBCA injection [Gadovist, Bayer, 1,0 mmol/ml]) showing a non-mass-enhancement in the right breast [orange arrow]. The examination was performed using a 3T MRI (Vida, Siemens Healthineers, Germany) with an 18CH breast coil (Siemens Healthineers, Germany). The examination revealed a concomitant aortic aneurysm of 6 cm size in the ascending aorta (white arrow images B/C, transversal and reconstructed coronal view respectively). Further clarification was recommended and followed by a contrast enhanced computed tomography (CT, Siemens X.ceed, contrast agent: 110 ml Imeron 350, Bracco) confirming the aneurysm of the ascending aorta (lower images in the right panel side). Biopsy of the BI-RADS 4 lesion finding revealed an extended apocrine metaplasia without DCIS components. Aortic aneurysm repair was not [yet] performed (at the time of the publication).

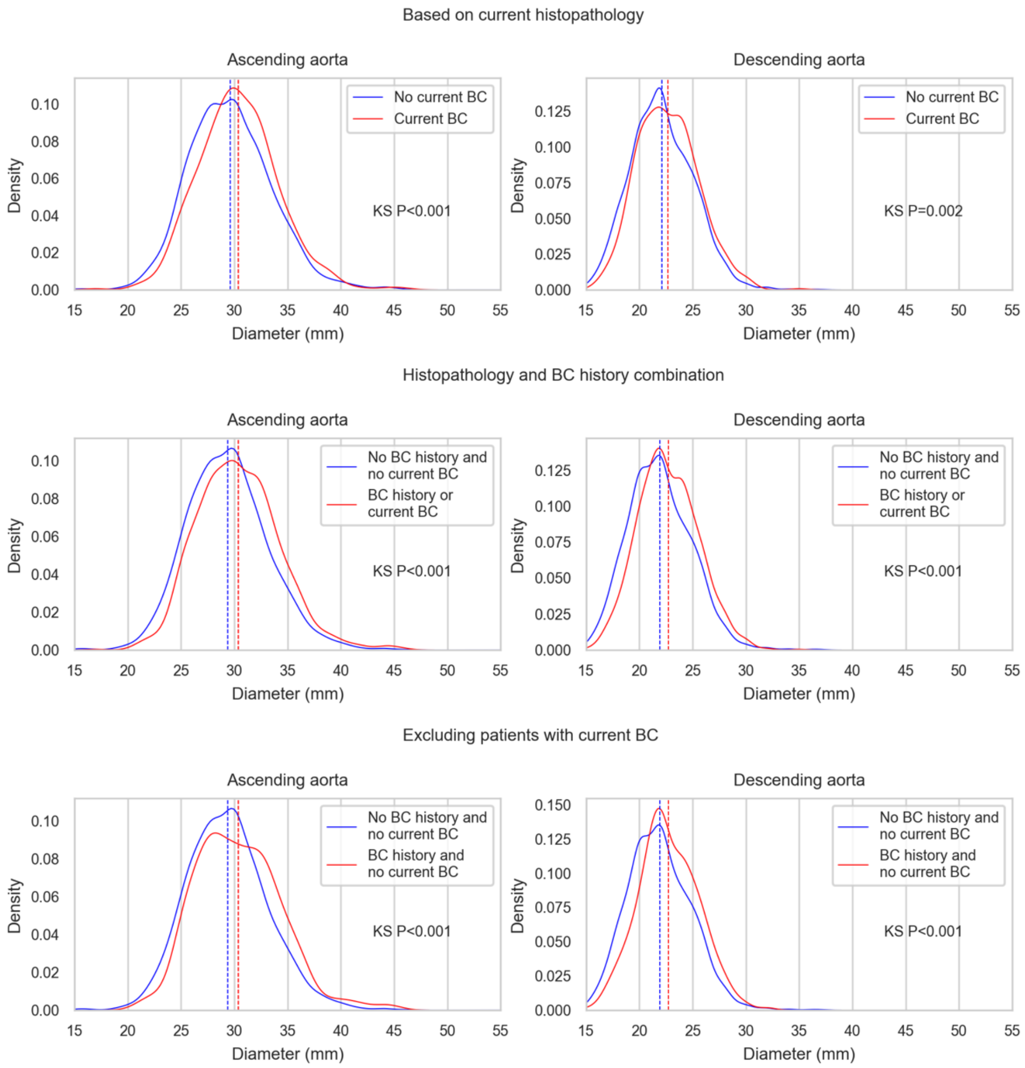

Supplementary Figure 4: Differences of the maximum thoracic aorta diameter in women with breast cancer (BC) or history of breast cancer in the Erlangen test dataset with n=3232 breast MRI examinations, presented with Kernel Density Estimate (KDE) plots.

Examinations of women with breast cancer presented a higher average maximum diameter and a higher odds ratio of presenting with a thoracic aortic aneurysm (OR=2.14, CI: [0.51, 8.97], first row). The effect was present when history of breast cancer was also considered (OR=2.29, CI: [0.55,9.61], second row) and when patients with current breast cancer were excluded and only history of breast cancer was analyzed (OR=1.91, CI: [0.32,11.48], third row). Statistical analysis was performed using the Kolmogorov–Smirnov (KS) test. However, due to the retrospective study design and the origin of the data, this finding might be biased by the selection of patients that underwent a clinically indicated breast MRI. Further, given that the confidence intervals are wide due to the low prevalence of aneurysms and the fact that there was an absence of a truly non-clinical control population, no definite conclusions can be drawn regarding potential underlying causes for this finding, warranting additive studies.

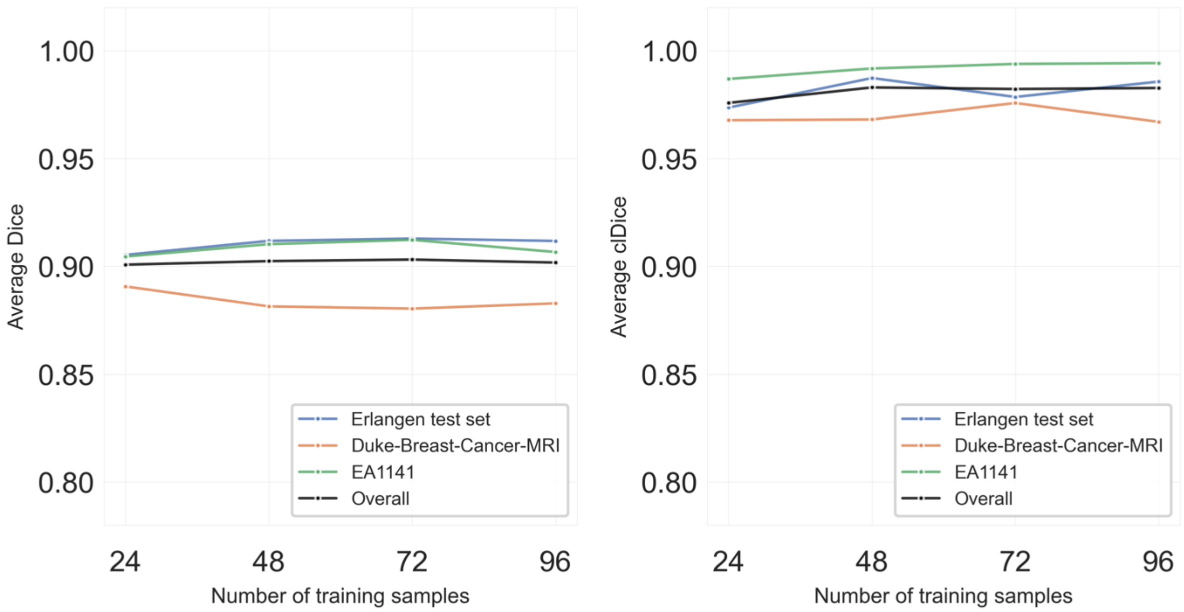

Supplementary Figure 5: Effect of the number of training samples on segmentation performance (Dice and clDice).
The segmentation performance appears stable, indicating no direct need for more training samples. Performed across all ground truth segmentations (n=28 for the Erlangen test set, n=20 for Duke-Breast-Cancer-MRI, and n=20 for EA1141).

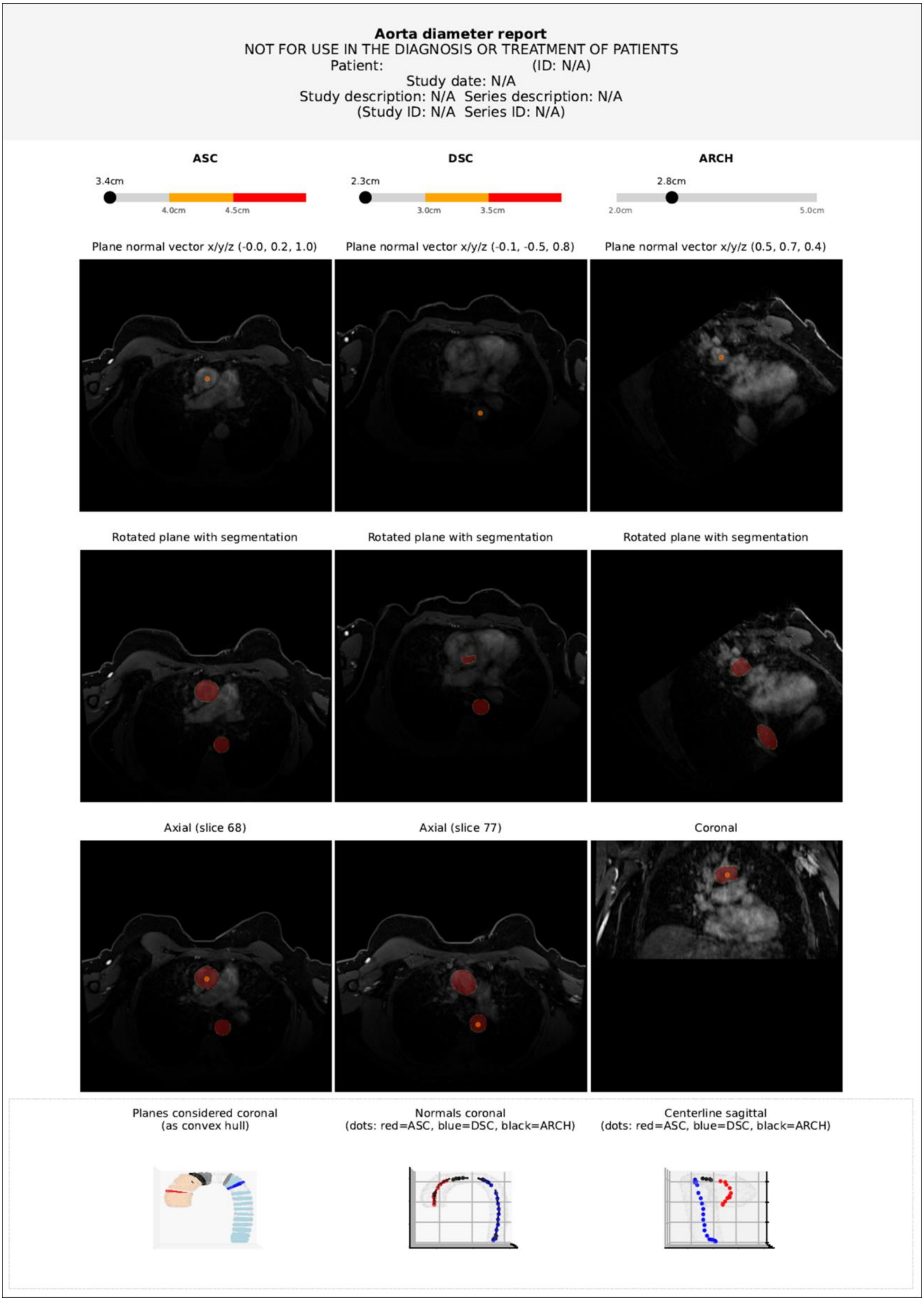

Supplementary Figure 6: Example PDF report produced by the ANN pipeline.

Supplementary Table 1: MRI device vendor and model breakdown for included studies.				
	**In-house dataset (training)**	**Erlangen test dataset**	**DUKE**	**EA1141**
**Breast MRI examinations**	96	3232	922	903
**MRI device vendor**				
**Siemens Healthineers**	96	3232	294	612
MAGNETOM Aera	0	313	0	231
MAGNETOM Avanto	0	0	179	22
MAGNETOM Espree	0	0	0	212
MAGNETOM Free.Max	1	4	0	0
MAGNETOM Sola	0	2	0	0
MAGNETOM Vida	95	2208	0	0
MAGNETOM Skyra	0	0	57	8
MAGNETOM Skyra fit	0	704	0	1
MAGNETOM Symphony	0	0	0	9
N/A	0	1	0	0
MAGNETOM Trio	0	0	1	0
MAGNETOM TrioTim	0	0	57	3
MAGNETOM Verio	0	0	0	126
**GE Medical Systems**	0	0	628	233
DISCOVERY MR450	0	0	0	24
DISCOVERY MR750	0	0	0	41
DISCOVERY MR750w	0	0	0	33
Optima MR450w	0	0	98	18
SIGNA EXCITE	0	0	10	0
SIGNA Explorer	0	0	0	3
SIGNA HDx	0	0	272	0
SIGNA HDxt	0	0	248	110
SIGNA Pioneer	0	0	0	4
**Philips Medical Systems**	0	0	0	58
Achieva	0	0	0	58
Abbreviations: MRI=Magnetic Resonance Imaging; N/A=not available.				

Supplementary Table 2: T1-weighted sequence acquisition protocols used in the Erlangen dataset.							
**Model name**	**Magnetic field strength (T)**	**Sequence**	**Matrix**	**FoV (mm)**	**TE (ms)**	**TR (ms)**	**Slice thickness (mm)**
Aera	1.5	DIXON	384 × 323 to 448 × 376	379 - 410	2.39 - 4.77	6.49	1.5 - 1.6
Aera	1.5	FLASH (w/o fat saturation)	384 × 336 to 448 × 394	340 - 429	4.77	7.7 - 7.72	1.7 - 2.1
Avanto	1.5	FLASH (w/o fat saturation)	448 × 331	340 - 399	4.78	7.58 - 8.32	1.5 - 1.8
Skyra fit	3.0	FLASH (with DIXON)	448 × 358 to 448 × 385	360 - 429	2.46	5.51 - 5.97	1.5 - 1.9
Skyra fit	3.0	VIBE (with DIXON)	448 × 385	359 - 399	2.46	5.97	1.5 - 1.7
Skyra fit	3.0	FLASH (with fat saturation)	448 × 385	359 - 399	2.46	6.04	1.5 - 1.7
Sola	1.5	FLASH (with DIXON)	448 × 358	379	4.77	6.5	1.8
Vida	3.0	FLASH (with DIXON)	448 × 358 to 448 × 385	379	2.46	5.41	1.5 - 1.7
FreeMax	0.55	FLASH-3D	390 x 390	390	2.3	5.6	2.0
Abbreviations: T=Tesla (referring to the field strength of the MRI device); TE=echo time;TR=time to repetition; ms=millisecond; mm=millimeter; FoV=field of view.							

Supplementary Table 3: Technical analysis and error rates of the ANN pipeline.				
	**Erlangen test dataset**	**Duke**	**EA1141**	**Overall**
**Breast MRI examinations (n)**	3232	922	903	5057
**Patients (n)**	2258	922	480	3660
**Analysis success of the ANN pipeline for thoracic aorta assessments**				
Technically successful ANN plane analysis of any aorta segment, amongst:	3131 (96.9%)	892 (96.7%)	852 (94.4%)	4875 (96.4%)
Ascending thoracic aorta (AA)	3089	749	844	4682
Descending thoracic aorta (DA)	3112	843	623	4578
Aortic Arch	2250	581	733	3564
Error rate of the ANN pipeline	0.56%	4.56%	1.55%	1.46%
Mis-segmentation related	0.28%	2.06%	1.00%	0.73%
Error in diameter determination despite adequate segmentation	0.28%	2.49%	0.55%	0.73%
Mean deviation of ANN derived diameters from manual diameters*	1.52 mm (CI: [1.45, 1.58])	1.52 mm (CI: [1.39, 1.65])	1.52 mm (CI: [1.32, 1.72])	1.52 mm (CI: [1.46, 1.58])
(Human inter-rater variation of aorta diameter measurements)	1.61 mm (CI: [1.50, 1.72])	1.69 mm (CI: [1.47, 1.91])	1.72 mm (CI: [1.48, 1.95])	1.64 mm (CI: [1.55, 1.73])
Ascending thoracic aorta (AA)	1.44 mm	1.48 mm	1.42 mm	1.45 mm
Descending thoracic aorta (DA)	1.59 mm	1.55 mm	1.65 mm	1.59 mm

*Analyzed in a random subset of n=1060 ascending or descending aorta measurements (from n=560 breast MRI examinations randomly chosen from the different independent test datasets).

Abbreviations: ANN=Artificial Neural Network; AA=ascending thoracic aorta, DA=descending thoracic aorta; mm=millimeter; CI= confidence interval

Supplementary Table 4: Analysis of additive clarification examinations and associated costs caused by the ANN depending on PPV thresholds chosen (either using TTE or CE-CT)

	PPV	*NNC per detected aneurysm*	*Costs for TTE per detected aneurysm*	*Costs for CE-CT per detected aneurysm*
PPV 1 - Ascending thoracic aorta (AA)	20.8%	*n=4.8*	*$464 x 4.8 = $2,227.2*	*$246.13 x 4.8 = $1,181.24*
PPV 2 - Ascending thoracic aorta (AA)	55.5%	*n=1.8*	*$464 x 1.8 = $835.2*	*$246.13 x 1.8 = $443.03*
PPV 3 - Ascending thoracic aorta (AA)	100%	*n=1*	*$464 x 1 = $464*	*$246.13 x 1 = $246.13*
PPV 1 - Descending thoracic aorta (DA)	6.67%	*n=15*	*$464 x 15 = $6,960*	*$246.13 x 15 = $3,691.95*
PPV 2 - Descending thoracic aorta (DA)	N/A	N/A	N/A	N/A
PPV 3 - Descending thoracic aorta (DA)	N/A	N/A	N/A	N/A

Cost estimations according to Wei et al., 2023 (for TTE) and Zimmerman et al., 2021 (for CE-CT). Abbreviations: PPV1=all cases exceeding dilatation thresholds; PPV2=all cases reaching aneurysm thresholds minus a 2mm safety margin; PPV3=all cases exceeding aneurysm thresholds; NNC=number of patients needing to undergo additive examinations for clarification of their finding in the breast MRI examination; TTE=transthoracic echocardiography; CE-CT= contrast enhanced computed tomography; N/A=not available.
